# Post-traumatic hydrocephalus after decompressive craniectomy: a multidimensional analysis of clinical, radiological, and surgical risk factors

**DOI:** 10.1007/s10143-025-03673-0

**Published:** 2025-06-21

**Authors:** Sérgio Miguel Fernandes Romualdo, Tareq Adnan Juratli, Ilker Eyüpoglu, Gabriele Schackert, Markus Dengl, Markus Prem, Mido Max Hijazi, Kerim-Hakan Sitoci-Ficici

**Affiliations:** Department of Neurosurgery, Faculty of Medicine, Technische Universität Dresden, University Hospital Carl Gustav Carus, Dresden, Germany

**Keywords:** Decompressive craniectomy, Traumatic brain injury, Post-traumatic hydrocephalus, Hydrocephalus

## Abstract

Decompressive craniectomy is a key treatment for refractory intracranial pressure after severe traumatic brain injury (TBI). Post-traumatic hydrocephalus (PTH) occurs in 7.6–36% of cases, and early diagnosis significantly improves rehabilitation outcomes. This retrospective study analyzed risk factors for shunt-dependent PTH in 126 TBI patients (93 men, 33 women, median age 53 years). Patients were divided into those requiring shunts and those who did not. Clinical and radiological characteristics, including volumetric measurements and surgical techniques, were assessed using SPSS^®^ Statistics 25. The incidence of shunt-dependent PTH was 27%. Multivariate analysis identified significant risk factors: advanced age at craniectomy (*p* = 0.008; OR 1.048), traumatic subarachnoid hemorrhage in the basal cisterns (*p* = 0.015; OR 7.545), post-traumatic ischemic infarcts (*p* = 0.003; OR 5.319), transcalvarial brain herniation (*p* = 0.012; OR 5.543), subdural hygroma (*p* = 0.004; OR 8.131), and progression of contusion hemorrhages (*p* = 0.013; OR 4.386). Operative parameters did not show statistical significance. Neurological outcomes in shunt patients, assessed via the modified Rankin Scale and Extended Glasgow Outcome Scale, were significantly worse than in non-shunt patients (mRS > 3, GOS-E < 5, *p* = 0.001–0.011). Our findings suggest that subarachnoid hemorrhage in the cisterns, advanced age, hygromas, ischemic infarcts, transcalvarial herniation, and contusion hemorrhage progression are independent risk factors for shunt-dependent PTH. Additionally, shunt placement was linked to poorer neurological outcomes.

## Clinical trial number

Not applicable.

## Introduction

Traumatic brain injury (TBI) affects approximately 69 million individuals worldwide annually, underscoring its significance as a major public health challenge with a broad range of severe and lasting consequences [15, 26]. The pathophysiology of TBI is complex and involves both initial and secondary damage phases that can result in irreversible functional loss, emphasizing the need for prompt and effective intervention [36, 52].

Among the critical interventions for managing severe TBI, decompressive craniectomy (DC) has been identified as a lifesaving procedure aimed at reducing elevated intracranial pressure. However, the DECRA trial has highlighted that while effective in mitigating intracranial pressure, the procedure’s impact on functional outcomes is multifaceted, necessitating careful consideration of patient selection [12, 21, 25].

The occurrence of post-traumatic hydrocephalus (PTH) following TBI, especially after interventions, such as DC, adds another layer of complexity to patient recovery. PTH, marked by altered cerebrospinal fluid (CSF) dynamics, contributes to a range of adverse outcomes including decreased consciousness, cognitive dysfunction, gait disturbances, and incontinence [49, 55, 58]. With an incidence rate reported between 2.1% and 34%, the variability in PTH development post-TBI highlights the necessity of a nuanced approach to management and treatment [32, 46]. This condition underscores the critical balance of hydrostatic and osmotic forces in CSF production and absorption, challenging traditional treatment paradigms, and signaling the need for advancements in our understanding and approaches to care [2, 4, 10, 37].

Given the profound impact of PTH on the quality of life of TBI survivors, this study aimed to delineate the incidence of PTH in patients who have undergone DC for TBI and identify associated risk factors. By investigating predictive factors and the relationship between surgical techniques and PTH development, our research aims to enhance clinical decision-making, improve patient outcomes, and ultimately contribute to the improvement of care for individuals affected by TBI and its sequelae.

## Materials and methods

**Fig. 1:**
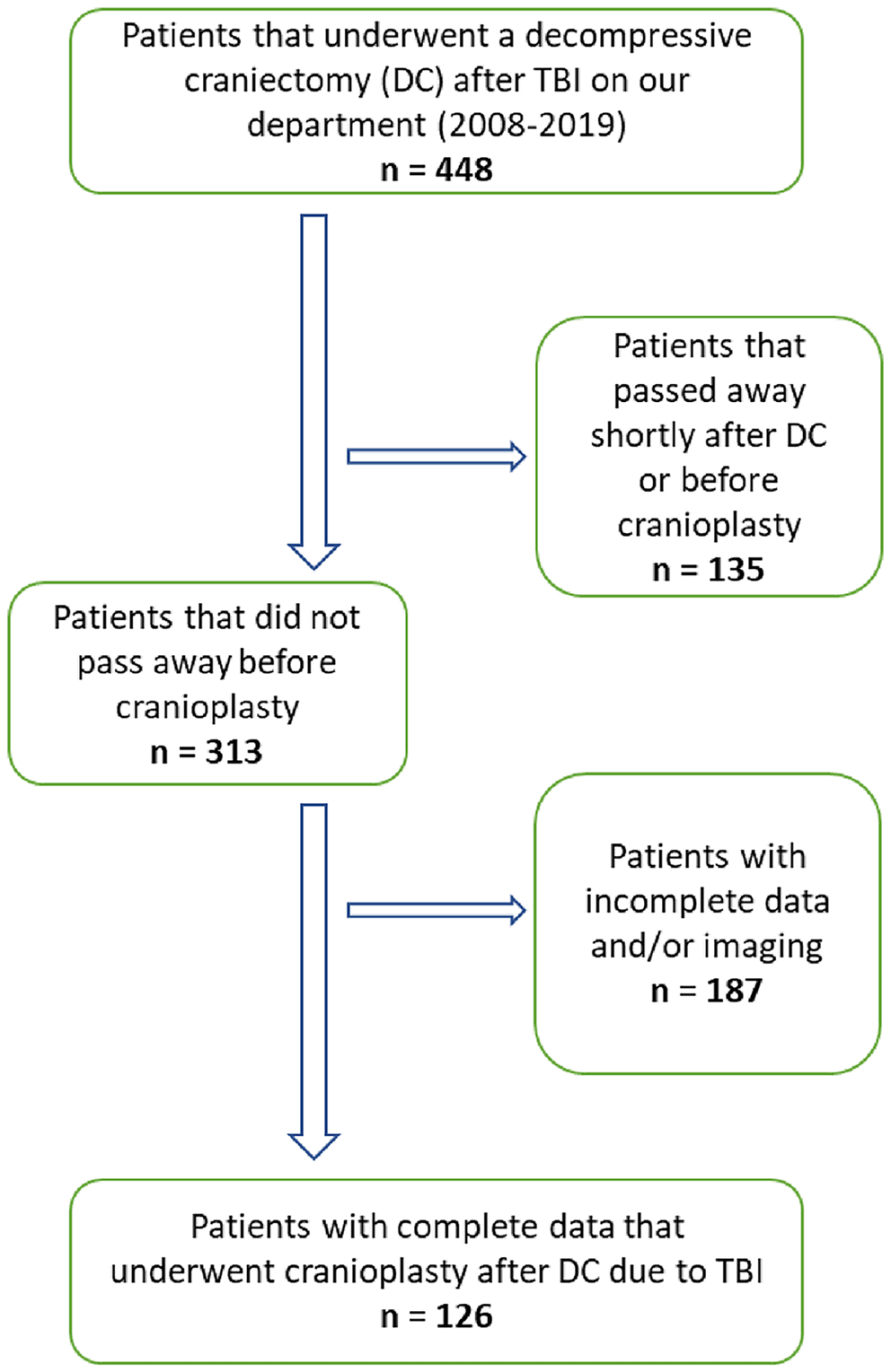
Flow diagram of patients´ inclusion/exclusion

This study, approved by the ethics committee of *Technische Universität Dresden*, was a retrospective observational analysis conducted at the University Hospital Dresden, a level I trauma center. We included 126 patients aged 18–84 years who underwent DC after TBI between 2008 and 2019. Patients were eligible if pre-operative CT scans and comprehensive post-operative clinical and imaging data were available. Exclusions applied to patients who had DC performed elsewhere, were younger than 18 years, lacked adequate clinical and imaging follow-up data, or died before undergoing cranioplasty (CP) (Fig. 1).

### Decompressive craniectomy: indication and technique

DC was indicated for patients with intracranial mass lesions exhibiting midline shift, performed either immediately or delayed, based on clinical and imaging evidence of deterioration and unmanageable intracranial pressure (ICP). Some patients received external ventricular drains (EVD) for CSF drainage in cases of significant intraventricular hemorrhage (IVH) or dilated ventricles. The unilateral surgical approach ensured a minimum extension of 12 cm in the sagittal plane, including sphenoidal and temporal osteoclastic widening, to alleviate the pressure on the temporal lobe and midline structures.

### Post-traumatic hydrocephalus diagnosis

PTH was diagnosed in patients with ventriculomegaly and clinical deterioration. Evan´s index was determined retrospectively as a quantitative measure of ventriculomegaly in all patients. Affected patients are treated with CSF shunting, typically via ventriculoperitoneal shunts. In this manner, for the purposes of this study and its data analysis and in line with its retrospective design, a patient was classified as having developed PTH if they underwent shunt implantation.

### Study parameters and data collection

Patient demographics, neurological status, outcome, and imaging data (CT) were analyzed, and the detailed findings are summarized in (Table 1) and (Table 2). Imaging was reviewed using Agfa IMPAX EE (Agfa Healthcare, Bonn, Germany). Subsequent semiautomatic segmentation for 3D reconstruction and volumetric measurements of traumatic hemorrhages and the craniectomy were performed using BrainLab Elements (BrainLab AG, Munich, Germany). To calculate the craniectomy volume, the post-operative skull volume was subtracted from the pre-operative volume to provide a quantitative measure of the bone removed during surgery.

**Table 1:** Results summary 1

	Sample *n* (%) / median	non-Shunt *n* (%) / median	Shunt *n* (%) / median	Univariate analysis (*p*)	Multivariate analysis (*p*)
**Clinical characteristics**					
Age at the time of DC (years)	53	52	59	**0**,**041**	**0**,**008**
Male sex	93 (73,8%)	66 (71,7%)	27 (79,4%)	0,495	
GCS < 13	98 (77,8%)	69 (75,0%)	29 (85,3%)	0,334	
Anisocoria	41 (32,5%)	27 (29,4%)	14 (41,2%)	0,284	
No pupil light reaction	40 (31,7%)	28 (30,4%)	12 (35,3%)	0,668	
Anticoagulants/Antiaggregant	31 (24,6%)	21 (22,8%)	10 (29,4%)	0,488	
**Pathoanatomical findings**					
SDH	117 (92,9%)	85 (92,4%)	32 (94,1%)	1	
SDH-Breite (mm)	11,35	11,1	11,9	0,998	
SDH Bilateral	10 (7,9%)	8 (8,7%)	2 (5,9%)	0,728	
IH-SDH	47 (37,3%)	32 (34,8%)	15 (44,1%)	0,407	
IH-SDH-Breite (mm)	5,1	5	6,3	0,366	
Contusion hemorrhage	88 (69,8%)	61 (66,3%)	27 (79,4%)	0,192	
Contusion hemorrhage frontal	61 (48,4%)	42 (45,7%)	19 (55,9%)	0,323	
Contusion hemorrhage temporal	47 (37,3%)	31 (33,7%)	16 (47,1%)	0,214	
EDH	21 (16,7%)	14 (15,2%)	7 (20,6%)	0,59	
IVH initial	14 (11,1%)	7 (7,6%)	7 (20,6%)	0,055	
IVH (overall)	28 (22,2%)	17 (18,5%)	11 (32,4%)	0,146	
IVH (firstly seen in the FU)	14 (11,1%)	10 (10,9%)	4 (11,8%)	1	
tSAH on the convexity > 5 mm	25 (19,8%)	14 (15,2%)	11 (32,4%)	**0**,**044**	
tSAH in the cisterns	13 (10,3%)	5 (5,4%)	8 (23,5%)	**0**,**006**	**0**,**015**
MS pre-DC (mm)	9	9	9,05	0,506	
TCH in cCT-FU	82 (65,1%)	54 (58,7%)	28 (82,4%)	**0**,**02**	**0**,**012**
PTCI in cCT-FU	55 (43,7%)	31 (33,7%)	24 (70,6%)	**< 0**,**001**	**0**,**003**
sHYG in cCT-FU	86 (68,3%)	57 (62,0%)	29 (85,3%)	**0**,**017**	**0**,**004**
CL-sHYG in cCT-VLK	21 (16,9%)	10 (10,9%)	11 (33,3%)	**0**,**006**	
IHH in cCT-VLK	19 (15,1%)	10 (10,9%)	9 (26,5%)	**0**,**047**	
Contusion progress pre-DC	38 (30,2%)	27 (29,4%)	11 (32,4%)	0,828	
Contusion progress cCT-FU	33 (26,2%)	17 (18,5%)	16 (47,1%)	**0**,**002**	**0**,**013**
Evan´s Index > 0,3	54 (42,9%)	21 (22,8%)	33 (97,1%)	**< 0**,**001**	
Total	126 (100%)	92 (73%)	34 (27%)		

**Table 2:** Results summary 2

	Sample *n* (%) / median	non-Shunt *n* (%) / median	Shunt *n* (%) / median	Univariate analysis (*p*)	Multivariate analysis (*p*)
**Surgical parameters and timing**					
Bifrontal DC	22 (17,5%)	14 (15,2%)	8 (23,5%)	0,297	
DC Volume (cm^3^)	134,7	133,1	138,1	0,199	
Distance DC-ML (mm)	21,35	22	20,4	0,495	
Distance DC-ML ≤ 25 mm	86 (68.3%)	60 (65.2%)	26 (76.5%)	0.284	
Distance DC-ML ≤ 20 mm	59 (46.8%)	42 (45.6%)	17 (50%)	0.692	
Time to DC	1:28 h	1:38 h	1:09 h	**0**,**013**	
Primary DC	90 (71,4%)	61 (66,3%)	29 (85,3%)	**0**,**045**	
Time from DC to CP (d)	87	88	82,5	0,332	
Time of DC to Shunt-Implantation (d)	85				
EVD	13 (10,3%)	3 (3,3%)	10 (29,4%)	**< 0**,**001**	
**Neurological outcome**					
mRS 4–5 at discharge post-DC	106 (84,1%)	72 (78,3%)	34 (100%)	**0**,**002**	
mRS 4–5 at discharge post-CP	81 (64,3%)	47 (51,1%)	34 (100%)	**< 0**,**001**	
GOS-E 1–4 at discharge post-DC	111 (88,1%)	77 (83,7%)	34 (100%)	**0**,**011**	
GOS-E 1–4 at discharge post-CP	82 (65,1%)	49 (53,3%)	33 (97,1%)	**< 0**,**001**	
**Volume metric parameters**					
Volume of SDH pre-DC (all) (cm3)	60,75	51,6	78,5	0,556	
Volume SDH pre-DC (> 0) (cm3)	68,3	62,4	78,9	0,774	
Volume Contusion pre-DC (all) (cm3)	0,83	0,81	1,4	0,437	
Volume Contusion pre-DC (> 0) (cm3)	5,3	4,7	5,7	0,874	
Volume Contusion post-DC (all) (cm3)	2,6	1,96	6,3	**0**,**04**	0,071
Volume Contusion post-DC (> 0) (cm3)	7,51	6,6	9,1	0,187	
Total	126 (100%)	92 (73%)	34 (27%)		

### Statistical analysis

Data were analyzed using SPSS for Windows Version 26 (IBM Deutschland GmbH, Ehningen, Germany). Independent predictors were identified using a multivariate logistic regression with stepwise backward elimination. Associations between variables were examined using the chi-square test, McNemar test, and Mann-Whitney U test. Statistical significance was set at *p* < 0.05.

## Results

### Population sample, demographics and shunt implantation rate

**Fig. 2:**
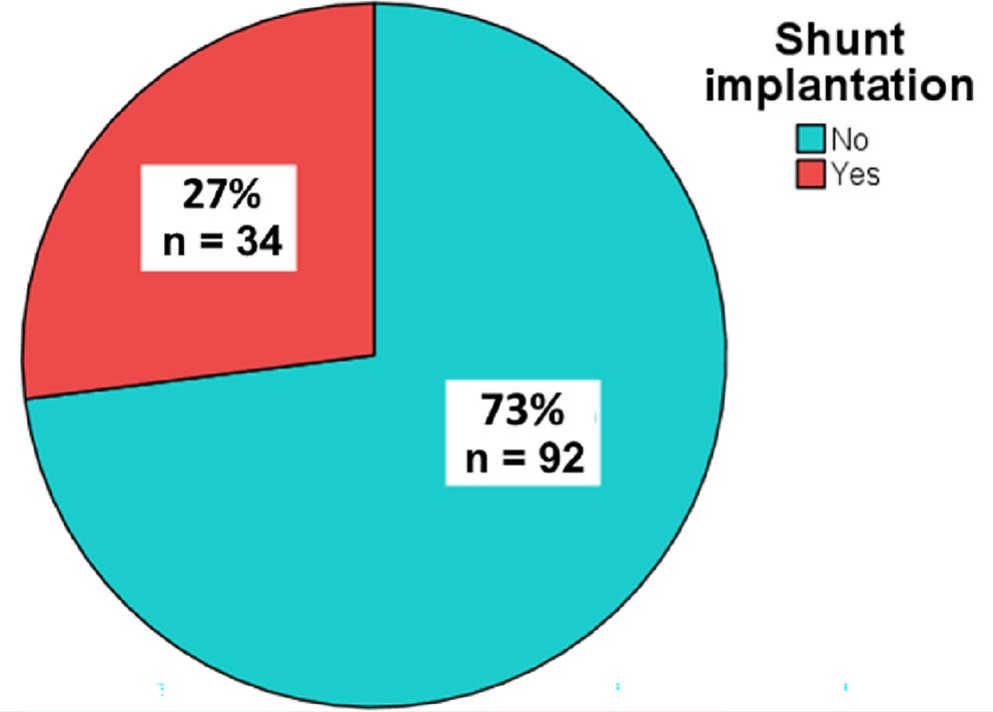
Shunt-implantation [Study population]

**Fig. 3:**
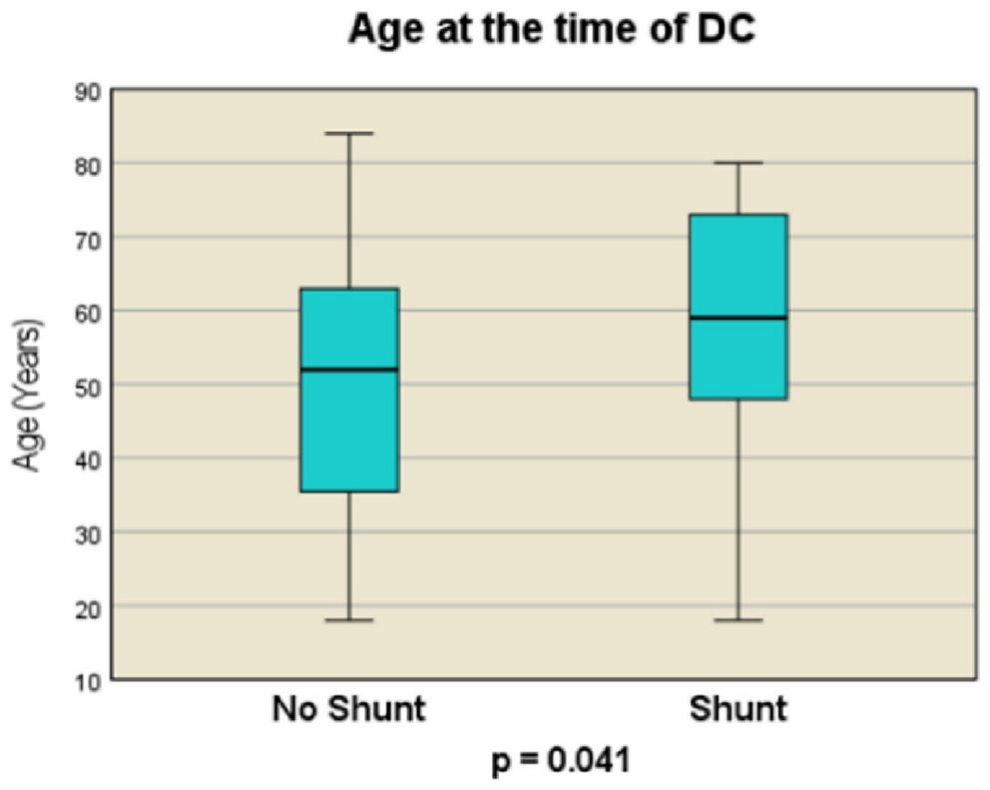
Age at the time of DC [no-Shunt vs. Shunt]

Our study included 126 patients who underwent DC followed by CP at the Department of Neurosurgery, University Hospital Dresden between May 2008 and April 2019. Of these, 93 patients were male (73.8%) and 33 were female (26.2%) (Fig. 2). The median age of the patients was 53 years (range, 18–84 years; IQR, 28 years). Shunt placement was performed in 34 (27%) patients (Fig. 3).

### Clinical characteristics

Patients with shunt were older than those without shunt (mean age, 59 years vs. 52 years; *p* = 0.041). Severe TBI Glasgow Coma Scale (GCS) scores of 3–8 were diagnosed in 63% of non-shunt and 61.8% of shunt patients, moderate TBI (GCS 9–12) in 12% and 23.5%, and mild (GCS 13–15) in 25% and 14.7% of patients, respectively. The analysis revealed no significant differences in the distribution of TBI severity (based on GCS) between the three GCS groups (*p* = 0.589). Similarly, comparison of the mild TBI cohort with the moderate/severe TBI cohort showed no significant disparity (*p* = 0.334). Initial anisocoria was diagnosed in 29.4% (*n* = 27) of the non-shunt cohort and in 41.2% (*n* = 14) of the shunt cohort, with no significant difference between the groups (*p* = 0.284). Anticoagulant or platelet inhibitor use (26.4% [*n* = 31] overall) did not significantly affect PTH levels (22.8% vs. 29.4%, respectively; *p* = 0.488).

### Intracranial pathologies and their association with PTH: an analysis of initial CT findings in TBI patients

Our study meticulously evaluated the initial cranial CT (cCT) scans of TBI patients to identify pathoanatomical features and their association with the subsequent necessity for shunt placement, indicative of PTH development.

#### Subdural hematoma

The incidence of convexity subdural hematoma (SDH) was notably high throughout the cohorts − 94.1% in shunt patients and 92.4% in non-shunt patients, with no significant variance observed (*p* = 1.0). The similarity in hematoma width between groups (median 12 mm, range 1–27 mm in non-shunt patients vs. median 11 mm, range 2–29 mm in shunt patients) further corroborates the lack of a direct link to PTH development. Bilateral SDH occurred in 5.9% (*n* = 2) of the shunt group and 8.7% (*n* = 8) of the non-shunt group, whereas interhemispheric SDH was present in 44.1% (*n* = 15) of the shunt cohort and 34.8% (*n* = 32) of the non-shunt cohort, with no significant correlation with PTH (Bilateral SDH, *p* = 0.728; Interhemispheric SDH, *p* = 0.407).

#### Contusion hemorrhage and epidural hematoma

Contusion hemorrhages were present in 79.4% (*n* = 27) of shunt patients compared to 66.3% (*n* = 61) of non-shunt patients, and epidural hematomas (EDH) were noted in 20.6% (*n* = 7) of the shunt cohort versus 15.2% (*n* = 14) of the non-shunt cohort. Neither pathology significantly predicted the development of PTH (contusion hemorrhage, *p* = 0.192; EDH, *p* = 0.59).

#### Intraventricular hemorrhage

**Fig. 4:**
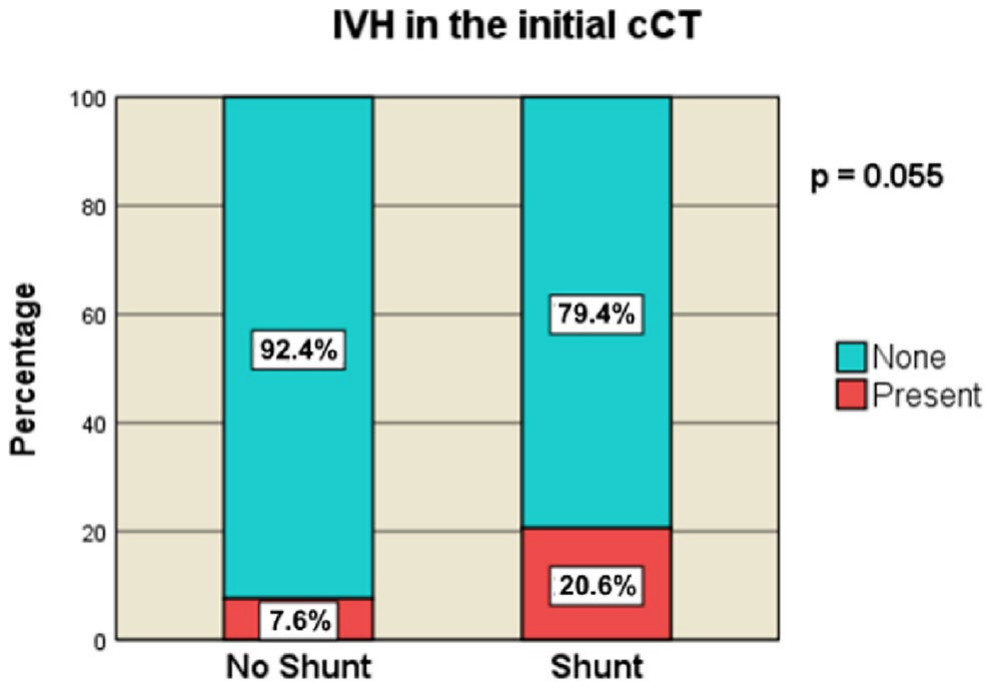
IVH in the initial cCT [no-Shunt vs. Shunt]

The occurrence of IVH in the initial CT scans demonstrated merely a trend towards an association with shunt necessity, occurring in 32.4% (*n* = 11) of the shunt cohort and 18.5% (*n* = 17) of the non-shunt cohort (overall IVH, *p* = 0.146; initial IVH, *p* = 0.055) (Fig. 4).

#### Traumatic subarachnoid hemorrhage

**Fig. 5:**
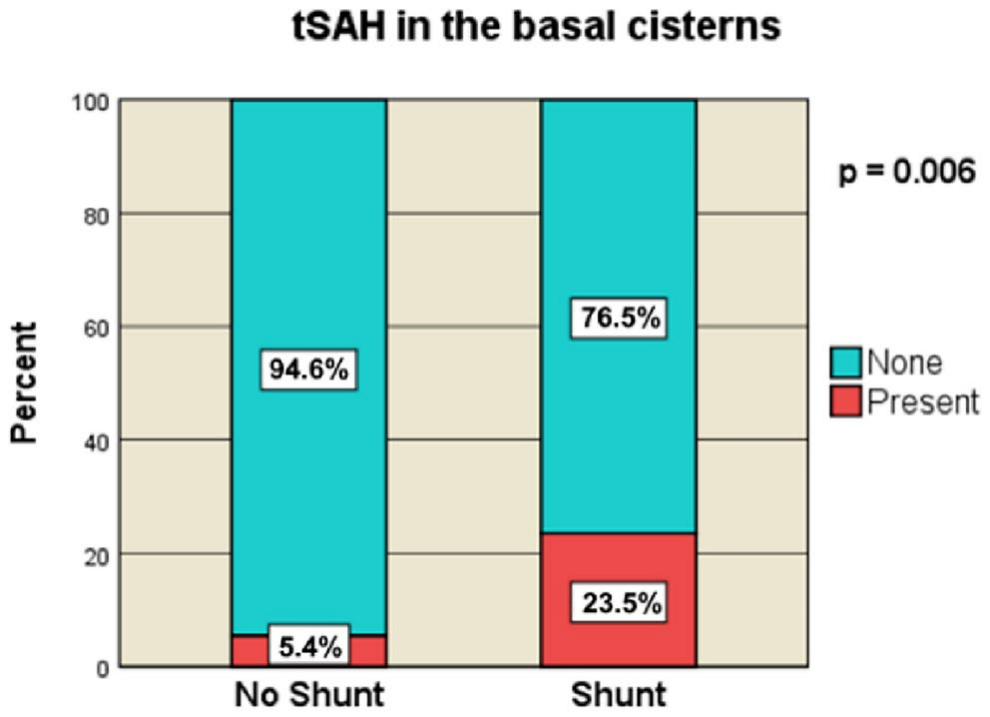
tSAH in the basal cisterns [no-Shunt vs. Shunt]

A significant association with PTH was observed for traumatic subarachnoid hemorrhage (tSAH), particularly in the shunt cohort with convexity tSAH > 5 mm in 32.34% (*n* = 11) and basal cistern tSAH in 23.5% (*n* = 8), compared with 15.2% (*n* = 14) and 5.4% (*n* = 5), respectively, in the non-shunt group (convexity tSAH > 5 mm, *p* = 0.044; basal cistern tSAH, *p* = 0.006) (Fig. 5).

#### Transcalvarial herniation and post-traumatic cerebral infarct

**Fig. 6:**
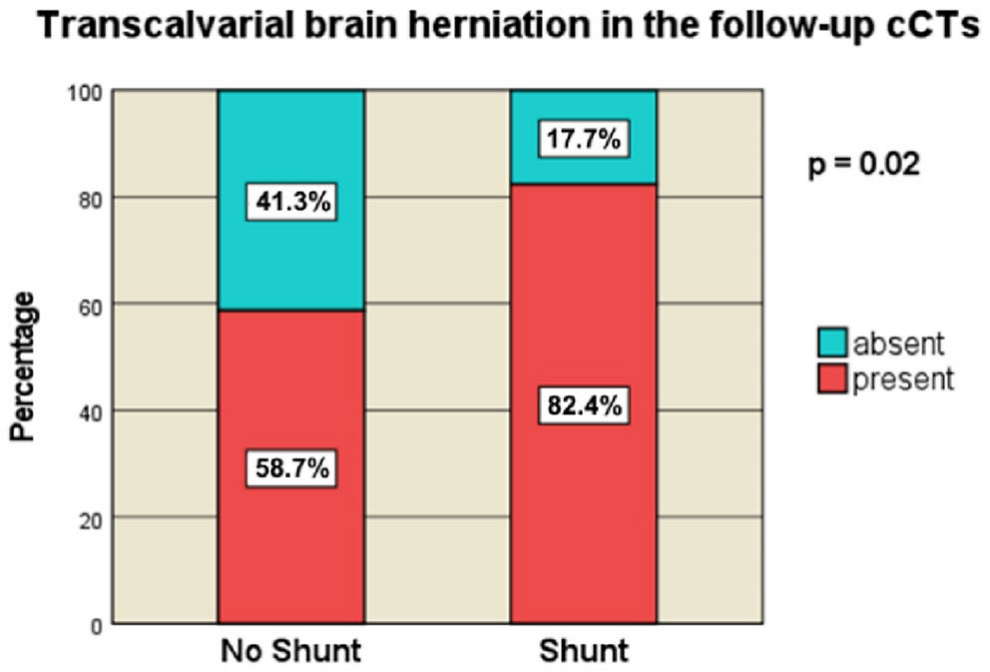
Transcalvarial brain herniation in the follow-up cCTs [no-Shunt vs. Shunt]

**Fig. 7:**
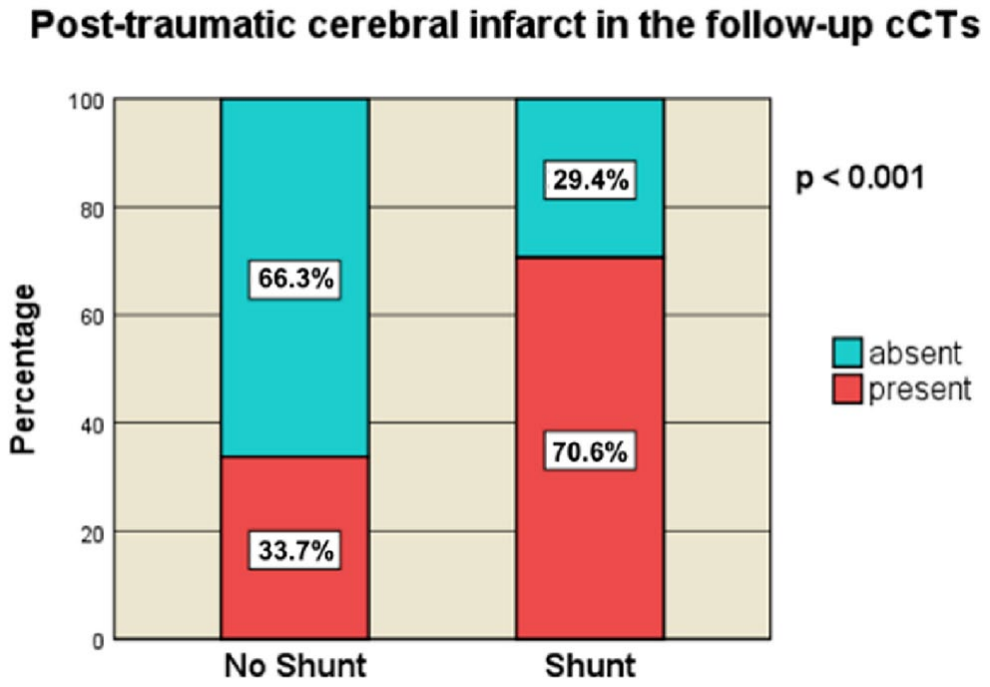
Post-traumatic cerebral infarct in the follow-up cCTs [no-Shunt vs. Shunt]

Transcalvarial herniation (TCH) was significantly more prevalent in the shunt cohort (82.4%, *n* = 28) than in the non-shunt cohort (58.7%, *n* = 54, *p* = 0.02) (Fig. 6). Post-traumatic cerebral infarct (PTCI) was also observed more frequently in the shunt group (70.6%, *n* = 24) than in the non-shunt group (33.7%, *n* = 31, *p* < 0.001) (Fig. 7). PTCI is defined as any ischemic lesion on the follow-up CTs. These findings indicate a direct correlation between these pathologies and the need for shunt placement in PTH management.

#### Subdural hygroma in the postoperative follow-up cCTs

**Fig. 8:**
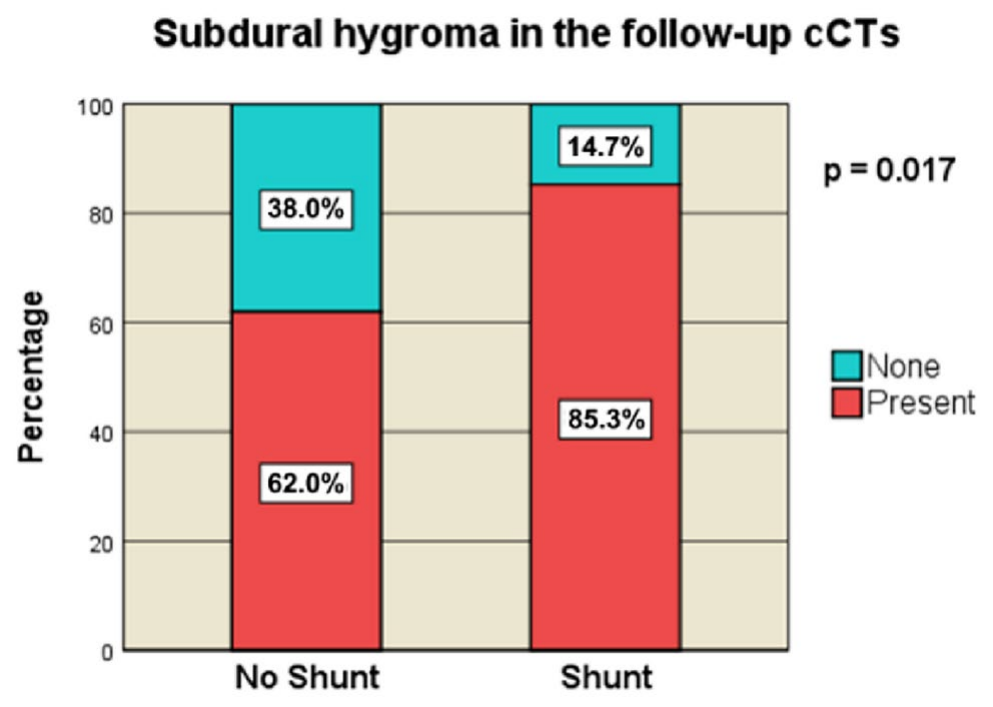
Subdural hygroma in the follow-up cCTs [no-Shunt vs. Shunt]

Subdural hygroma was significantly more common in the shunt group (85.3%, *n* = 29) than in the non-shunt group (62.0%, *n* = 57, *p* = 0.017) (Fig. 8). Contralateral subdural hygromas also showed a significant association with shunt placement, appearing in 33.3% (*n* = 11) of the shunt cohort versus 10.9% (*n* = 10) of the non-shunt cohort (*p* = 0.006). Interhemispheric subdural hygromas were significantly more prevalent in the shunt group (26.5%, (*n* = 9) than in the non-shunt group (10.9%, *n* = 10) (*p* = 0.047).

#### Midline shift prior to DC

Midline shift measurements prior to DC revealed no significant difference between the cohorts, with a median shift of 9.05 mm (range 0–25 mm) in the shunt group and 9 mm (range 0–26.1 mm) in the non-shunt group, highlighting comparable severity of brain displacement between patients regardless of shunt necessity (*p* = 0.506).

#### Progression of contusion hemorrhages in pre- and post-DC follow-up cCT

**Fig. 9:**
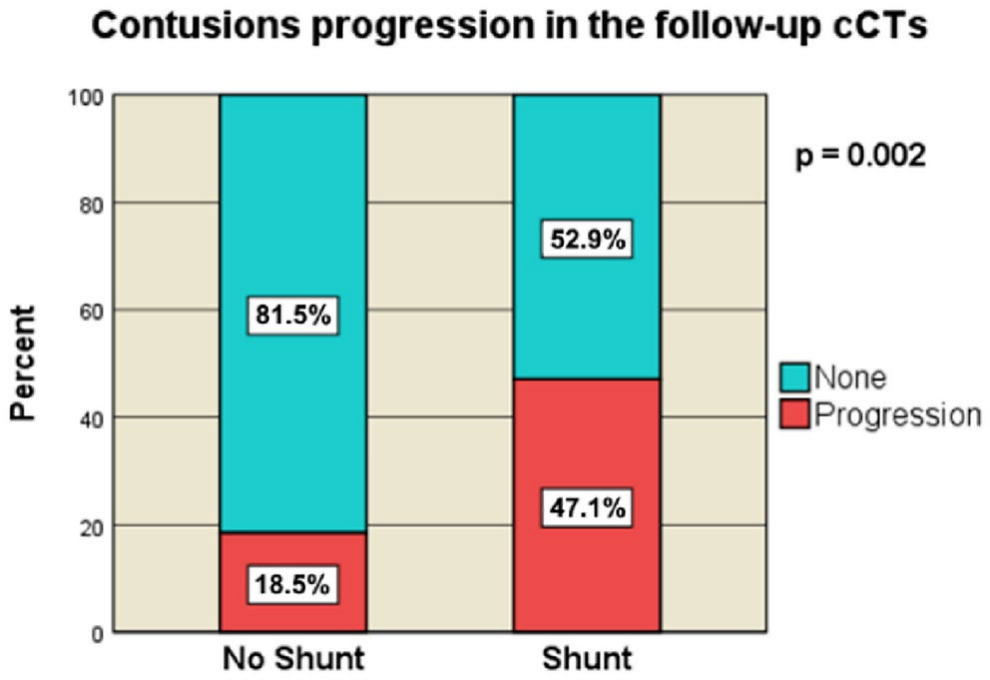
Contusion progression in the follow-up cCTs [no-Shunt vs. Shunt]

In the non-shunt group, 29.3% (*n* = 27) of patients exhibited progression of contusion hemorrhages on follow-up cCT prior to DC compared to 32.4% (*n* = 11) in the shunt group. This progression was not significantly associated with the need for shunt placement (*p* = 0.828). Post-DC, the incidence of contusion hemorrhage progression increased to 47.1% (*n* = 16) in the shunt cohort, which was significantly higher than that in the non-shunt group (18.5% (*n* = 17), indicating a significant correlation with shunt requirement (*p* = 0.002) (Fig. 9).

#### Evan´s index

The Evan´s index served as a benchmark for shunt necessity, along with clinical data. It was measured up to the last cCT control before CP or shunt implantation depending on the cohort. The highest value per patient was used for the analysis. The median Evan´s Index was 0.28 (range 0.14–0.50; IQR 0.04) in the non-shunt cohort and 0.45 (range 0.27–0.87; IQR 0.16) in the shunt cohort, showing a significant association with the necessity for shunt placement (*p* < 0.0001). Given the pathological threshold (0.30 for ventriculomegaly, further analysis with dichotomization at 0.30 reaffirmed this association: 77.2% (*n* = 21) in the non-shunt cohort had an Evans Index > 0.3, compared to 97% (*n* = 33) in the shunt cohort (*p* < 0.001).

### Surgical parameters and timing

#### Unilateral frontotemporoparietal vs. bifrontotemporal DC

Unilateral versus bifrontotemporal DC showed no significant difference in shunt necessity (*p* = 0.297), with bifrontotemporal DC performed in 15.2% (*n* = 14) of non-shunt patients and 23.5% (*n* = 8) of shunt patients.

#### Distance from craniectomy edge to midline

The median distance from the craniectomy edge to the midline was 22 mm (range 0–54.7 mm; IQR, 16.0 mm) in the non-shunt cohort and 20.4 mm (range, 0–43 mm; IQR, 12 mm) in the shunt cohort, with no significant difference noted (*p* = 0.495). Additionally, cut-off values were analyzed. In 65.2% (*n* = 60) patients of the non-shunt cohort had a craniectomy edge of ≤ 25 mm, to the midline, in comparison to 76.5% of the shunt patients (*n* = 26), yielding a non-significant p-value of 0.284. Decreasing the cut-off value to ≤ 20 mm showed as well, no difference (*p* = 0.692) between the cohorts: 45.6% (*n* = 42) non-shunt vs. 50% of the shunt patients (*n* = 17).

#### Timing from admission to DC

The median time from patient admission to the start of DC was 1 h 38 min (range 18–341 h 47 min; IQR 15 h 4 min) in the non-shunt group and 1 h 9 min (range 13–137 h 32 min; IQR 1 h 2 min) in the shunt cohort, showing a significant association with shorter admission-to-surgery times and shunt requirement (*p* = 0.013).

#### Primary vs. secondary DC

A primary DC was performed in 66.3% (*n* = 61) of the non-shunt group, compared to 85.3% (*n* = 29) in the shunt group, indicating a significant link with the need for a primary DC and shunt placement (*p* = 0.047).

#### Timing from DC to CP

The median interval between DC and CP was 88 days (range, 22–267 days; IQR, 42 days) in the non-shunt cohort and 82.5 days (range, 24–234 days; IQR, 40 days) in the shunt cohort, with no significant difference between the intervals (*p* = 0.332).

#### Placement of an EVD

EVD placement was significantly more frequent in the shunt cohort (29.4% (*n* = 10) than in the non-shunt group (3.3% (*n* = 3) in the non-shunt group (*p* < 0.001), aligning with its use in patients with cerebrospinal fluid circulation disturbances due to IVH or ventriculomegaly.

#### Craniectomy volume

The median craniectomy volume measured via BrainLab software was 133.1 cm³ (range 59.0–342.9 cm³; IQR 34.5 cm³) for the non-shunt cohort and 138.1 cm³ (range 88.3–305 cm²; IQR 23.8 cm³) for the shunt cohort. The difference between groups was not significant, indicating that craniectomy size did not influence shunt necessity (*p* = 0.199).

### Volume metrics and their correlation with shunt dependency

#### Pre-craniectomy convexity subdural hematoma volume

Initial scans showed a median SDH volume of 62.4 cm^3^ (range 1.38–226.8 cm^3^, IQR 83.8 cm^3^) for non-shunt and 78.9 cm³ (range 4.79–231.6 cm^3^, IQR 109.3 cm^3^) for the shunt group, again with no significant difference (*p* = 0.774), showing no direct link between SDH volume and shunt necessity.

#### Pre-craniectomy contusion hemorrhage volume

The pre-DC median contusion volume was 4.7 cm³ (range 0.05–94.1 cm^3^; IQR 12.9 cm^3^) for the non-shunt group and 5.4 cm³ (range 0.2–44.3 cm^3^; IQR 13.9 cm^3^) for the shunt group, without significant difference (*p* = 0.874), suggesting no correlation between contusion volume and shunt dependency despite slightly larger volumes in the shunt group.

#### Post-craniectomy contusion hemorrhage volume

Post-craniectomy, the median contusion volume was 6.6 cm³ (range 0.06–47.8 cm^3^; IQR 14.7 cm^3^) for non-shunt and 9.1 cm³ (range 0.17–83.5 cm^3^; IQR 20.9 cm^3^) for shunt patients, with this difference not reaching statistical significance (*p* = 0.187). An exploratory additional analysis, that included the complete patient sample and not only the patients that had a contusion hemorrhage showed a median volume of 1.96 cm³ (range 0–47.8 cm³; IQR 11.0 cm³) for non-shunt and 6.3 cm³ (range 0–83.5 cm³; IQR 16.0 cm³) for shunt groups, with a significant association found with shunt necessity (*p* = 0.04) within the whole cohort.

### Neurological outcomes and their correlation with shunt dependency

#### Modified Rankin Scale

Using the modified Rankin Scale (mRS), clinical outcomes were compared at two points: post-DC discharge and post-CP discharge. A score ≤ 3 was considered a favorable outcome, while a score > 3 indicated a poor outcome. At DC discharge, 21.7% of non-shunt patients had an mRS ≤ 3 compared to none (0%) in the shunt cohort, showing a highly significant difference (*p* = 0.002). At CP discharge, 48.9% of the non-shunt cohort had an mRS score ≤ 3, while all shunt cohort patients scored > 3, underscoring a highly significant difference (*p* < 0.001).

#### Glasgow outcome scale-extended

The Glasgow Outcome Scale-Extended (GOS-E) further compared clinical outcomes, with scores ≥ 5 indicating favorable outcomes. At DC discharge, 16.3% of non-shunt patients had a GOS-E ≥ 5, versus none (0%) in the shunt cohort, signifying a significant association with poor outcome in shunt-requiring patients (*p* = 0.011). At CP discharge, 46.7% of non-shunt patients had improved to GOS-E ≥ 5, a notable recovery compared with post-DC. Meanwhile, 97.1% of shunt patients continued to have poor outcomes (GOS-E < 5), with a very small fraction (2.9%) reaching a favorable outcome (*p* < 0.001).

#### Predictors of shunt necessity

Multivariate logistic regression revealed significant predictors of the development of post-traumatic hydrocephalus following DC (Table 3). Key factors included advanced age at surgery (OR = 1.048, *p* = 0.008, CI 1.012–1.085), presence of tSAH in basal cisterns (OR = 7.545, *p* = 0.015, CI 1.477–38.544), PTCI (OR = 5.319, *p* = 0.003, CI 1.767–16.007), TCH (OR = 5.543, *p* = 0.012, CI 1.449–21.199), subdural hygroma (OR = 8.131, *p* = 0.004, CI 1.942–34.048), and progression of contusion hemorrhages in follow-up cCTs (OR = 4.386, *p* = 0.013, CI 1.360-14.148).

**Table 3:** Predictors of shunt necessity

Independent Variable	OR	Significance	CI 95%
Age at the time of DC	1.048	0.008	1.012–1.085
tSAH in the basal cisterns in the initial cCT	7.545	0.015	1.477–38.544
PTCI in follow-up cCTs	5.319	0.003	1.767–16.007
TCH in follow-up cCTs	5.543	0.012	1.449–21.199
Subdural hygroma in follow-up cCTs	8.131	0.004	1.942–34.048
Progression of contusions in follow-up cCTs	4.386	0.013	1.36-14.148

**Fig. 10:**
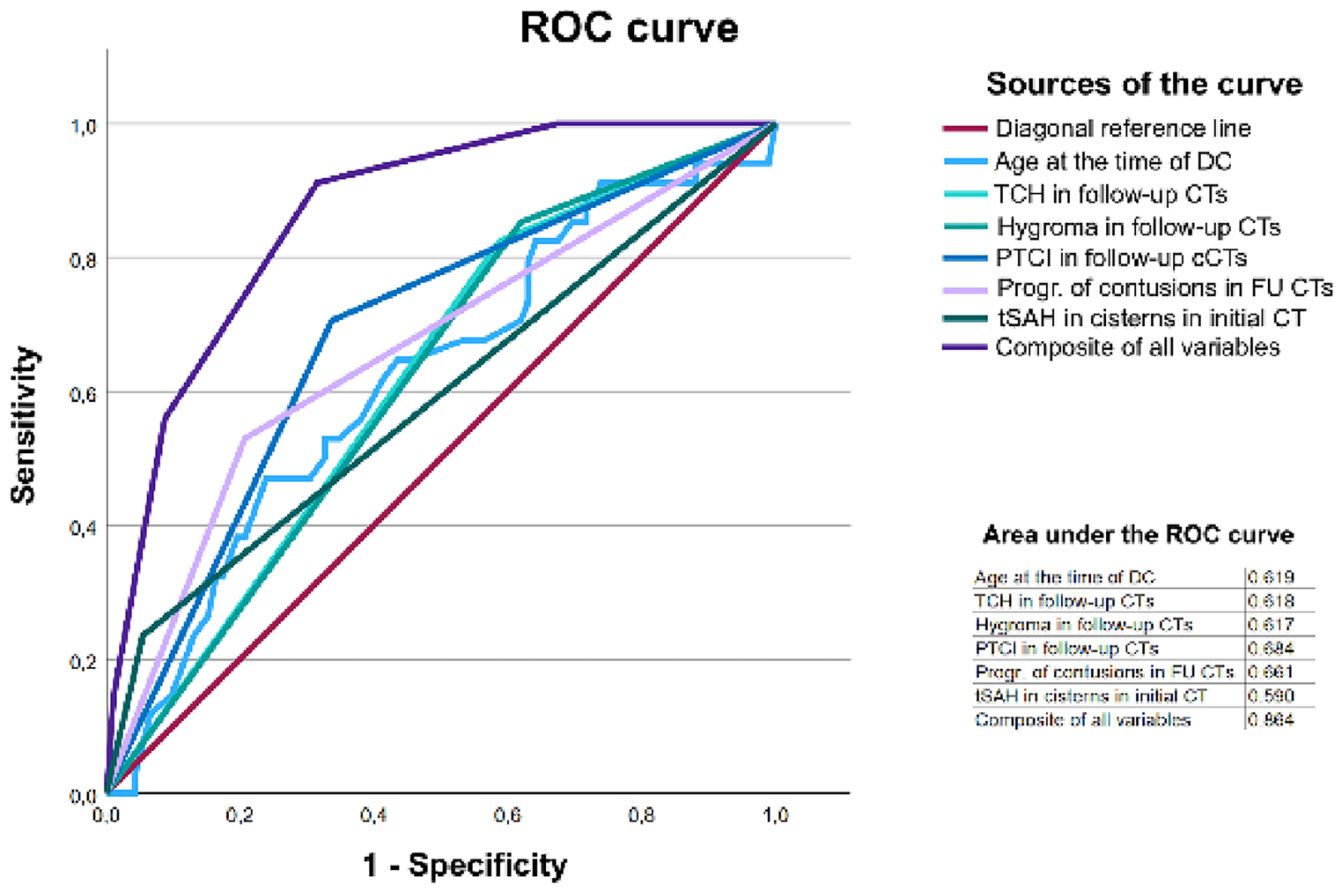
ROC curve. For the creation of composite curve, each individual variable was given the value of one. For the age parameter (metric), a cut-off value of ≥ 55 years old was used

Additionally, we performed a ROC analysis. While the individual predictors demonstrated poor to fair discriminatory ability, the composite model comprising all six highly significant independent variables achieved an AUC-ROC of 0.864. The findings are detailed in (Fig. 10)

## Discussion

We designed a retrospective study on patients with TBI who underwent decompressive craniectomy to identify predictors and risk factors associated with the development of PTH after DC. Our detailed volumetric measurements of brain contusions, acute SDH, and craniectomy dimensions were aimed at determining their potential roles in PTH development. Key predictive factors identified included advanced age at surgery, presence of subarachnoid hemorrhage in basal cisterns, post-traumatic ischemic brain infarcts, transcalvarial herniation, and subdural hygroma.

### Incidence and patient demographics

Our findings revealed a PTH incidence rate of 27%, aligning with the variability reported in the literature ranging from 2.1 to 34% [17, 23, 56]. We identified a significant correlation between advanced age and the development of PTH after DC, consistent with findings from other studies [24, 48]. Contrasting studies have shown a link between younger age and PTH [35, 53], while others found no significant age-related differences [11, 14, 16, 23, 28, 29, 39, 46]. Research indicates that age-related changes in cerebral blood flow and hydrodynamics, including significant reductions in total arterial cerebral blood flow and cerebrospinal fluid dynamics, may affect cerebrospinal fluid dynamics, potentially influencing the development of PTH post-DC [5, 47, 57]. No significant associations were found between sex and PTH development in our study, reflecting similar outcomes reported in the literature [11, 16, 23, 24, 28, 29, 39, 46, 48, 53, 56].

### Severity of TBI

Several studies have linked a low initial GCS with the development of PTH following DC, with multivariate analysis identifying it as an independent variable [16, 23, 24, 45, 46, 48]. Di et al. and Marmarou et al. have highlighted that low GCS scores often reflect severe brain tissue damage, potentially leading to significant disruptions in cerebrospinal fluid flow and absorption, culminating in hydrocephalus [16, 33]. Contrary to these findings, our study revealed no significant correlation between initial GCS scores and PTH, a discrepancy that may be attributable to cohort-specific biases or the nuanced nature of the GCS, which does not linearly correlate with cerebrospinal fluid circulation issues and TBI severity [6, 41, 50]. The complexity of GCS interpretation is compounded by factors such as the variability of the timing post-injury, differences in sedation practices, and assessment inconsistencies across different clinical settings [6, 42]. Regarding pupil status, we found no association with PTH occurrence, consistent with some reports [29, 35, 39, 48], but in disagreement with others [16, 23, 24, 46], suggesting variability that could be attributed to inconsistencies in the measurement of pupil status across different studies.

### Impact of TBI sequelae on PTH

#### Intraventricular hematoma

In this analysis, the presence of an IVH in initial cCT scans showed a trend towards the development of PTH following DC, although without statistical significance, suggesting its potential as an early PTH predictor. Subsequent examinations over time did not establish a correlation. Vedantam et al. arrived at similar conclusions [53]. However, these findings contrast with other studies [16, 24, 28, 29]. Some authors discussed that IVH might lead not only to acute hydrocephalus by obstructing CSF pathways or by impairing CSF absorption due to blood degradation products, but also to chronic PTH [16, 51]. Further analysis, specifically regarding the possible role of IVH-volume, may be warranted.

#### Traumatic subarachnoid hemorrhage

A tSAH of greater than 5 mm in the convexity correlated strongly with PTH, and tSAH was a predictor of PTH in our study, supported by findings from Nasi et al. and Vedantam et al. [35, 53], although some other studies found no correlation [16, 23, 24, 28, 29, 39]. Cerebrospinal fluid dynamics are governed by hydrostatic and osmotic forces, with capillaries primarily facilitating CSF production and absorption due to their large surface area [9]. Kuo & Huang noted that fibrosis in the subarachnoid space after a aneurysmatic SAH could lead to chronic hydrocephalus [30]. Obstruction of CSF flow by blood in basal cisterns, particularly beyond the Foramen Luschka and Magendie, can also induce hydrocephalus [8].In our study, SAH appeared to play a more prominent role in the development of PTH than IVH. While IVH may contribute more significantly to the onset of acute obstructive hydrocephalus, SAH seems to be more closely associated with the progression to chronic malresorptive hydrocephalus.

#### Post-traumatic cerebral infarct

We identified PTCI in follow-up cCT scans as a predictor of PTH after DC, consistent with [24, 48], though contrary results were noted in other studies [16, 29]. The pathophysiological link between PTCI and PTH is multifaceted and probably centered around the disruption of normal CSF dynamics. Cerebral infarct cause reduced regional cerebral blood flow and microangiopathy [20]. Peña and colleagues suggest that a reversal of interstitial fluid flow back into the parenchyma and reduced tissue elasticity and stiffness, often consequent to diminished cerebral perfusion and microangiopathy, pave the way to ventricular dilation associated with communicating hydrocephalus [20, 40]. This might explain the link between PTCI and PTH, but the precise causal relationship remains unclear [24]. Su et al. hypothesized that severe TBI, leading to substantial mass effect, could favor the development of post-operative cerebral infarcts [24]. The potential link between SAH-induced vasospasms and cerebral infarcts also remains speculative, necessitating further studies to explore this hypothesis. In addition, PTCI may maintain an elevated intracranial pressure despite decompressive measures.

#### Subdural hygroma after DC

The presence of subdural hygroma, regardless of location, was identified as a risk factor for PTH after DC. This finding is supported by previous studies [23, 29, 46, 48], although some researchers have observed only a trend without clear statistical significance [24, 28, 35, 53]. As for the hygroma’s location in our study, subdural hygromas contralateral to the DC were associated with PTH, consistent with other studies [24, 28, 48]. Ki et al. and Su et al. identified contralateral subdural hygroma as an independent risk factor for shunt-dependent PTH after DC [28, 48], whereas Di et al. reported no correlation [16]. Interhemispheric hygromas also show an association in our study, in accordance to other reports that identified a significant correlation [24, 27, 28, 35, 48, 53], with three considering it a risk factor for PTH development after DC [27, 35, 53].

The causal link between subdural hygroma and PTH as well as its pathogenesis remains unclear. Electron microscopy studies indicate no vacant space between the subarachnoid and dural layers, instead showing a complex interface made of the arachnoidal barrier and dural border cell layers [19, 44]. These layers are structurally supported by significant collagen in the dura, but minimally in the border cell layer, which is susceptible to mechanical stress (e.g., shear forces from traumatic brain injury). The subarachnoid trabeculae, protected by the arachnoid barrier, which is held together by desmosomes and tight and gap junctions, suggest that shearing forces might disrupt this layer, leading to a potential pathology [19]. Kaen et al. hypothesized a two-phase model for the formation of interhemispheric hygromas following severe traumatic brain injury, beginning with an increased intracranial pressure and shifting brain tissue across the falx cerebri [27]. Following DC, this shift could reverse, creating a suction effect and expanding the interhemispheric space, potentially causing a mechanical or inflammatory blockage of CSF outflow (rebound phase). Over time, as suggested by Waziri et al., disturbances in intracranial pressure dynamics due to craniotomy might decrease CSF outflow (hydrodynamic phase), simplifying the likely multifactorial processes involving additional inflammatory factors [27, 54].

#### Trancalvarial brain herniation after DC

In this study, we identified TCH as a predictor of PTH levels after DC. This association is supported by the results of Kaen et al. and Silva Neto et al., who also highlighted the role of TCH in the emergence of PTH post-DC [27, 46]. This link may stem from the severe impact of trauma on cerebral blood flow regulation, leading to hyperemia. This hyperemic state likely responds to the increased cerebral perfusion pressure seen after DC, resulting in a pronounced mass effect exerting a significant force on the decompressive site.

#### Contusion volume and progression

In the present investigation, we quantified the volume of contusion hemorrhages using semiautomatic segmentation (Brainlab^®^ software). The analysis revealed no association between pre-operative contusion volume and PTH development after DC. In contrast, the volumes of contusions observed post-operatively showed a significant correlation with PTH when considering the entire cohort, including those without contusions. This disparity might be due to the population diversity in our cohort and the localization and severity of contusions. Notably, the progression of contusion hemorrhages on follow-up skull CT scans after craniectomy was a predictor of PTH after DC. This suggests that both the presence and progression of contusions reflect the severity of TBI and may indicate an adverse coagulation state or suboptimal management. Future studies are needed to explore whether a disturbed coagulation state is directly related to the development of PTH following craniectomy.

### Does surgical timing or technique play a role?

#### Unilateral vs. bifrontotemporal DC

Our analysis found no correlation between unilateral versus bifrontotemporal DC and PTH development. Our results are consistent with those of other studies [23, 29]. Conversely, some studies have shown that bilateral craniectomy predisposes patients to PTH more than unilateral craniectomy [16, 28, 35, 45]. Di et al. identified bifrontal DC as an independent risk factor for the development of PTH post-DC [16], suggesting variations in study designs, patient numbers, and definitions of PTH contribute to these discrepancies.

#### Proximity of craniectomy to the midline

No association was found between the proximity of craniectomy to the midline and the occurrence of PTH post-DC in our study cohort. This lack of correlation mirrors the findings of four other studies [16, 23, 29, 46], while some others observed only a slight tendency [24, 28, 56]. Conversely, De Bonis et al. observed a significant association and recommended performing the craniectomy more than 25 mm from the midline as a protective measure against PTH (De Bonis et al., 2010) [14]. It was suggested that a craniectomy near the midline may disrupt mechanical forces on the veins during the diastolic phase, potentially increasing venous outflow, enhancing extracellular fluid absorption, reducing brain tissue volume, and causing ventricular enlargement [3, 14]. In cases of TCH near the midline, it may compress the sagittal bridging veins essential for brain venous drainage and CSF absorption [13]. Recent studies show CSF absorption occurs near the dural venous sinus, focusing interest on the “parasagittal dural space,” which is being actively researched [1, 22, 43]. A craniectomy close to the midline could change flow and pressure in the PDS, potentially contributing to PTH development. However, a review of craniectomy proximity to the midline and PTH found no statistically significant trends and could not recommend specific guidelines [7]. An alternative hypothesis of CSF physiology and hydrodynamics has gained increasing traction in recent years. Several studies have challenged the traditional view that the choroid plexus is the primary source of CSF production, instead suggesting that CSF is continuously produced and reabsorbed throughout the entire CSF system [10, 37]. According to this model, known as the Bulat-Klarica-Orešković hypothesis, CSF dynamics are primarily governed by hydrostatic and osmotic gradients between the CSF, interstitial fluid, and the extensive capillary network—now considered the principal sites of CSF formation and absorption. This view is further supported by the discovery of the glymphatic system, which describes perivascular pathways facilitating the distribution and clearance of substances through the CNS parenchyma [9, 38]. Together, these models promote a more integrated and decentralized understanding of CSF physiology. They may also explain our study’s findings and similar observations in other studies—of no clear association between the proximity of the DC to the midline and alterations in CSF dynamics.

#### Craniectomy size

In this analysis, the size of the craniectomy did not appear to influence the development of PTH post-DC, consistent with findings from some studies [14, 46, 48], but contrasting with others that noted an association [18, 24, 28, 29]. The size was identified as an independent risk factor, suggesting that DC might impact CSF dynamics and trigger inflammatory processes around the Pacchionian granulations [18, 28]. Methods for calculating craniectomy size vary, often without clear documentation. Fotakopoulos et al. measured the craniotomy area by multiplying the distances between frontal-parietal and temporal-parietal bone edges, resulting in a rectangular area [18]. De Bonis et al. used the formula A = “π[(d/2)² + h²]”, defining “d” as the longest anterior-posterior distance and “h” as the perpendicular distance to the lateral dural flap, based on Münch et al.‘s method [14, 34]. Our study uniquely measured craniectomy volume through semiautomatic segmentation using BrainLab^®^ software.

#### Timing of the DC

Our analysis identified an association between a shorter interval from admission to surgery and the occurrence of PTH post-DC, contrasting with some published studies [11, 16, 23, 24, 35, 39, 48]. Noteworthy is the heterogeneity in the categorization of ‘early’ versus ‘late’ craniectomy in these studies, often using a threshold of 6 h. Our analysis considered timing as a continuous variable. In addition to this continuous variable component, we divided our cohort into two groups: patients who underwent primary or secondary DC. A primary DC was performed in cases where a DC was indicated from the time of first admission, whereas a secondary DC was performed when the initial radiological and clinical parameters did not clearly necessitate a DC but later became indicated due to clinical or radiological deterioration. Our results suggest that primary DC is significantly associated with PTH. This suggests a correlation with the severity of TBI rather than a direct causal relationship, indicating that the timing likely reflects both the urgency of the intervention and the pathological severity evident in imaging and clinical assessments.

#### Timing of the CP

No correlation was found between the timing of CP and the development of PTH post-DC in our study, confirming findings from previous research [16, 23, 28, 29]. Contrary studies suggested delayed CP as an independent factor in the development of shunt-dependent PTH, suggesting that earlier CP (2 and 3 months) could reduce the incidence [35, 39]. Nevertheless, our study did not replicate these findings.

#### EVD-placement

In the present study, EVD was placed in 13 (10.3%) patients, with 3.3% (*n* = 3) in the non-shunt cohort and 29.4% (*n* = 10) in the shunt cohort. Early dilation of ventricles in the context of acute cerebrospinal fluid accumulation led to the placement of EVDs, demonstrating an association with the development of PTH after DC.

### Neurological outcomes

We identified a definitive correlation between poor neurological outcomes and development of post-traumatic hydrocephalus (PTH) following DC. The evaluations were recorded at discharge after DC (3–4 weeks post-DC) and then at discharge after CP (10–16 weeks post-DC), demonstrating a strong significance for poor outcomes. Comparison with existing literature further validates our results, underscoring the effect of PTH on functional recovery of patients [16, 24, 27, 35, 39, 48], and one using the mRS [23]. Discrepancies noted in other studies depict the complexity of TBI outcomes [11, 53]. These discrepancies may stem from variations in study methodologies, patient demographics, or definitions of outcomes and highlight the need for a standardized approach to evaluating and reporting TBI outcomes.

### Limitations

The retrospective nature of our study is one of the limitations. Retrospective analyses are susceptible to selection and information bias, which could affect the accuracy of the data collected and the conclusions drawn. One notable limitation of this study was the exclusion of patients without a complete data set or who succumbed to their TBI. Although this may appear overly stringent, the decision to include only those with complete datasets was made to ensure a high level of data comparability across the cohort. However, this approach may have introduced a selection bias that is difficult to fully account for. As data was collected from a single level I trauma center, the findings may not be generalizable to other settings with different patient demographics, clinical practices, or healthcare facilities. This limitation affects the external validity of our results, potentially restricting their applicability to broader populations. Although our sample size is appropriate for initial observations, larger, multicentric studies might provide a more robust analysis and help validate our findings across various settings. The study population was comprised of a wide range of age groups. The variability in follow-up durations among participants may lead to underestimation or overestimation of the incidence of PTH. Patient management adhered uniformly to our practice standards, which did not change during the study period. Semi-automatic segmentation was performed by only one examiner experienced in the segmentation of imaging data, reducing interobserver variability. The diagnosis of PTH was based on imaging and clinical data. Additional tests, such as infusion studies to assess CSF outflow resistance or MRI-based CSF flow measurements, were not performed in our cohort. Such tests could provide valuable insights into the pathophysiology of PTH following DC. Although we didn’t use CSF infusion tests, these tests are highly valuable as they could offer a more detailed understanding of CSF dynamics, helping to identify specific mechanisms contributing to PTH development and potentially guiding therapeutic strategies. Lalou et al. investigated CSF dynamics in patients with non-acute post-traumatic ventriculomegaly (PTV) using infusion testing. Compared to idiopathic normal pressure hydrocephalus patients, those with PTH showed lower intracranial pulse amplitude, indicating reduced compliance. Resistance to outflow was higher in PTH patients selected for shunting, suggesting its utility in treatment decisions. Imaging alone was often inconclusive, reinforcing the value of CSF dynamic assessment in diagnosing and managing PTH [31].

## Conclusions

Our study delineated the complex nature of post-traumatic hydrocephalus development following decompressive craniectomy following traumatic brain injury. We identified several predictors that influence the likelihood of post-traumatic hydrocephalus, including advanced age, presence of subarachnoid hemorrhage, and specific sequelae of traumatic brain injury and possibly decompressive craniectomy itself, such as progression of contusion hemorrhages, subdural hygroma, transcalvarial herniations, and post-traumatic ischemic strokes. Interestingly, no direct correlations were found between surgical parameters such as the volume of the craniectomy and distance from the midline and the development of post-traumatic hydrocephalus, indicating that the occurrence of post-traumatic hydrocephalus may be more influenced by patient-specific pathophysiological factors than by the surgical technique itself. Significantly, there was a strong association between the development of post-traumatic hydrocephalus and poor neurological outcomes. This underscores the profound impact of post-traumatic hydrocephalus on patient recovery and highlights the importance of monitoring these predictors in clinical practice. Our use of advanced volumetric segmentation significantly enhanced the precision of our data, supporting these findings and suggesting that future research should continue to refine diagnostic and treatment strategies to better manage brain injury.

## Data Availability

Raw data from individual cases cannot be shared to protect patient privacy. However, anonymized datasets and statistical analyses conducted in this study are available upon request. Researchers with methodologically sound proposals can contact the corresponding author for access. Proposals should be directed to sergio.romualdo@ukdd.de.

## Abbreviations

AUC: Area under the curve
CSF: Cerebrospinal Fluid
CT: Computer Tomography
cCT: cranial Computer Tomography
CP: Cranioplasty
DC: Decompressive Craniectomy
EDH: Epidural Hematoma
EVD: External Ventricular Drain
GCS: Glasgow Coma Scale
GOS-E: Glasgow Outcome Scale-Extended
ICP: Intracranial Pressure
IQR: Interquartile range
IVH: Intraventricular haemorrhage
MRI: Magnetic Resonance Imaging
mRS: modified Rankin Scale
MS: Midline Shift
OR: Odds Ratio
PTCI: Post-Traumatic Cerebral Infarct
PTH: Post-Traumatic Hydrocephalus
PTV: Post-Traumatic Ventriculomegaly
ROC: Receiver Operating Characteristic
SDH: Subdural Hematoma
sHYG: subdural Hygroma
TBI: Traumatic Brain Injury
TCH: Transcalvarial brain herniation
tSAH: traumatic Subarachnoid Haemorrhage
